# Molecular Hydrogen Modulates the Baroreflex Activity and Reduces the Vascular Adrenoreceptor Sensitivity to Phenylephrine and Lung Inflammation in Rats with Pulmonary Hypertension

**DOI:** 10.3390/biomedicines14030494

**Published:** 2026-02-24

**Authors:** Marina Artemieva, Larisa Kozaeva, Tatyana Kuropatkina, Khaidar Gufranov, Dmitrii Atiakshin, Natalia Medvedeva, Oleg Medvedev

**Affiliations:** 1 Faculty of Biology, Lomonosov Moscow State University, Leninskie Gory 1-12, 119234 Moscow, Russia; artemievamm@my.msu.ru (M.A.); medvedeva@mail.bio.msu.ru (N.M.); 2 Department of Pharmacology, Faculty of Medicine, Lomonosov Moscow State University, Lomonosovsky Prospect 27-1, 119991 Moscow, Russia; lali_k@mail.ru (L.K.); haidar@formed.ru (K.G.); medvedev@fbm.msu.ru (O.M.); 3 Laboratory of Medical Informatics and Healthcare Economics, Plekhanov University of Economics, Stremyannyi Pereulok 36, 115093 Moscow, Russia; 4 Research and Educational Resource Center for Immunophenotyping, Digital Spatial Profiling and Ultrastructural Analysis Innovative Technologies, RUDN University, Miklukho-Maklaya St., 6, 117198 Moscow, Russia; atyakshin-da@rudn.ru; 5 Laboratory of Experimental Pharmacology, National Medical Research Center of Cardiology Named After Ac Chazov E.I., Ak Chazova St., 15a, 121552 Moscow, Russia

**Keywords:** molecular hydrogen, antioxidants, monocrotaline-induced pulmonary hypertension, baroreflex, mast cells, tryptase, vascular reactivity, α_1_-adrenoceptor

## Abstract

**Background/Objectives:** Molecular hydrogen (H_2_), a natural antioxidant, can selectively reduce hydroxyl radicals and peroxynitrite without affecting signaling molecules such as H_2_O_2_ and NO. In addition, H_2_ can inhibit the synthesis of inflammatory cytokines. Human and animal studies have shown that the inhalation of H_2_ has a hypotensive effect. In this context, the aim of the present work was to study the effect of H_2_ on the baroreflex regulation of blood pressure in rats with experimental monocrotaline-induced pulmonary hypertension (MCT) in vivo and the effects of H_2_ on the reactivity of isolated rat aorta with MCT pulmonary hypertension to α_1_-adrenoceptor agonists in vitro. **Methods:** Experiments were performed on male Wistar rats with MCT pulmonary hypertension; animals were placed in plastic chambers aerated with atmospheric air at a rate of 4 L/min with O_2_ and CO_2_ control. Cages with the rats of the MCT-H_2_ and Control-H_2_ groups were ventilated with air containing 4% H_2_ twice daily for 2 h each. The MCT-Air and Control-Air groups breathed only atmospheric air. The duration of the experiment was 21 days. On day 20, blood pressure and heart rate (HR) were measured in awake animals and the baroreflex response to phenylephrine (PE) and nitroprusside (NP) was tested. In in vitro experiments, we studied the effect of adding H_2_ to the perfusion solution on the responsiveness of isolated aortic preparations from MCT and control rats to the α_1_-adrenoceptor agonist PE and the vasodilators NP and Acetylcholine. **Results:** When the effect of H_2_ on the baroreflex response to NP (4.5 μg/kg) was examined in awake rats, the increase in HR was 73.1 ± 16.7 beats/min in the MCT-Air group and 48.1 ± 10.2 beats/min in the MCT-H_2_ group (*p* < 0.01). In the Control-H_2_ and Control-Air groups, there was a trend towards a lower HR in the Control-H_2_ group, but the differences were not significant. No differences in HR response to PE administration were found between any of the experimental groups. Experiments on isolated aortic preparations from MCT rats showed that the addition of H_2_ to the perfusion medium resulted in a 30% reduction in the maximal response to PE compared with the MCT group without hydrogen (*p* < 0.01), and the potency of PE (EC_50_) decreased threefold (*p* < 0.05). There was a decrease in tryptase secretion, indicating an anti-inflammatory effect of H_2_. **Conclusions.** The results demonstrate that H_2_ inhalation was associated with an attenuated heart rate response to nitroprusside-induced hypotension and reduced vascular reactivity to phenylephrine in rats with pulmonary hypertension.

## 1. Introduction

Molecular hydrogen (H_2_) is colorless, odorless, the lightest chemical element, and it is not produced by eukaryotic cells. For this reason, H_2_ was long considered inert in mammalian cells. In 2007, Ohsawa I. et al. found that H_2_ can selectively reduce hydroxyl radicals (-OH) and peroxynitrite (ONOO-), which are very potent oxidants, thereby suppressing brain damage in ischemia/reperfusion (I/R) and stroke in a rat model [1]. According to the literature, H_2_ has a beneficial effect on the body with no side effects. Subsequent studies on cell cultures, animal experiments and clinical observations have demonstrated the preventive and therapeutic effects of H_2_ on various organs, including the cardiovascular system. A study in rats showed that the inhalation of H_2_ by the animals reduced ischemia–reperfusion injury to the myocardium by inhibiting oxidative stress and NLRP3-mediated (NOD-, LRR- and pyrin domain-containing protein 3) pyroptosis [2]. A protective effect of hydrogen gas has also been demonstrated in the study of cervical spinal cord ischemia–reperfusion injury [3]. The inhalation of hydrogen gas has been shown to reduce cardiac remodeling by reducing inflammation in rats with myocardial infarction [4] and in the lungs of animals with pulmonary hypertension [5].

Despite the large number of studies on the protective effect of H_2_ on the development of cardiac and cerebral pathologies, relatively few reports address its influence on systemic arterial pressure. One of these showed that daily 4 h inhalations of H_2_ led to a reduction in systolic blood pressure in elderly people (50–70 years old) diagnosed with arterial hypertension [6]. The authors suggest that in addition to changes in the activity of the renin-angiotensin system, a decrease in the activity of the sympathetic system is involved in this effect. In our previous study, investigating the effect of H_2_ on the development of monocrotaline-induced pulmonary hypertension (MCT-PH), it was shown that chronic inhalation of H_2_ reduces inflammation in the lung tissue and the value of mean blood pressure in the systemic circulation without affecting the main cardiovascular symptoms of the development of this disease [7]. Given the increasing use of H_2_ as a prophylactic and therapeutic agent in clinical practice, we investigated the effect of intermittent H_2_ inhalation on the severity of MCT-PH development and inflammation markers in lung tissue, as well as systemic blood pressure and the possible involvement of the sympathetic system in its regulation.

The intermittent inhalation protocol was selected based on previously published human [6] and animal [8,9] studies. A concentration of 4% H_2_ was used because such a level is strongly recommended, as it provides an effective concentration while eliminating life-threatening risks, ensuring patient and animal safety without compromising outcomes [10].

The MCT-induced pulmonary hypertension (MCT-PH) model was chosen because it does not significantly alter systemic blood pressure (BP) [11], allowing us to investigate the effects of molecular hydrogen independently of the mechanisms involved in systemic arterial hypertension. It is well established that the autonomic nervous system influences BP through central effects (e.g., modulation of heart rate and sympathetically controlled blood vessels) and peripheral effects (e.g., regulation of vascular tone) [12]. The arterial baroreflex serves as the primary regulator of autonomic neural activity (ANA): arterial baroreceptors continuously monitor changes in the arterial pressure and transmit signals to the brain, which dynamically adjusts ANA accordingly. Therefore, assessing baroreflex function is essential for understanding the role of the ANA in PH. Zimmer et al. demonstrated in a rat model of MCT-PH that MCT markedly increases SNA during the established stage of the disease [13]. Furthermore, implantable telemetry studies in rats showed that daily 1 h inhalation of 1.3% H_2_ suppresses sympathetic activity [8]. Accordingly, in the first series of experiments, we assessed heart rate responses to hypotensive and hypertensive agents to calculate baroreflex sensitivity—the key reflex responsible for BP stabilization. In the second series, we evaluated the effect of H_2_ on vascular reactivity by testing the response of isolated aortas from MCT-PH and control rats to vasoconstrictor and vasodilator agents.

Therefore, the aim and novelty of the present study were to investigate the effect of intermittent inhalation of 4% H_2_ on the degree of MCT-PH development, pulmonary inflammation and potential mechanisms of baroreceptor reflex involvement in the regulation of systemic blood pressure in experiments in vivo and on isolated rat aorta.

## 2. Materials and Methods

### 2.1. Animal Study

Inhalation of air with varying percentages of added H_2_ is currently widely used in experimental and clinical studies. Inhalation of a gas mixture with a hydrogen concentration of 2–4% has been shown to be safe [14].

#### 2.1.1. Animals

The study was performed on male Wistar rats weighing 180–220 g. The rats were kept under conditions of 12 h of light per day, with ad libitum access to food and water, and humidity and temperature controlled. Experiments were started not earlier than 7 days after the animals were received from the nursery. Only animals that successfully completed all surgical procedures (femoral artery and vein catheterization with exteriorized catheters) and all experimental stages were included in the final analysis. All procedures were performed in strict compliance with ethical standards, prioritizing minimizing animal distress. Experiments were conducted under the supervision of the Bioethics Committee of Lomonosov Moscow State University (MSU), which reviews and approves all protocols in accordance with the Recommendations of the Eurasian Economic Commission (EEC) Collegium dated 14 November 2023, No. 33, “On the Guidelines for Working with Laboratory (Experimental) Animals in Preclinical (Non-clinical) Studies”, as well as the national Russian standards GOST 33215-2014 “Housing and Care of Laboratory Animals (Equipment and Organization)” [15] and GOST 33216-2014 “Rules for Working with Laboratory Rodents and Rabbits” [16].

#### 2.1.2. MCT-PH Modeling

Pulmonary hypertension was induced by a single subcutaneous injection of MCT (*n* = 13) at a dose of 60 mg/kg dissolved in 60% ethanol (Sigma Aldrich, Germany, Darmstadt). The control animals (*n* = 18) received an equivalent volume of the MCT solvent (60% ethanol) without the active compound [17].

#### 2.1.3. Hydrogen Inhalation

**Figure 1:**
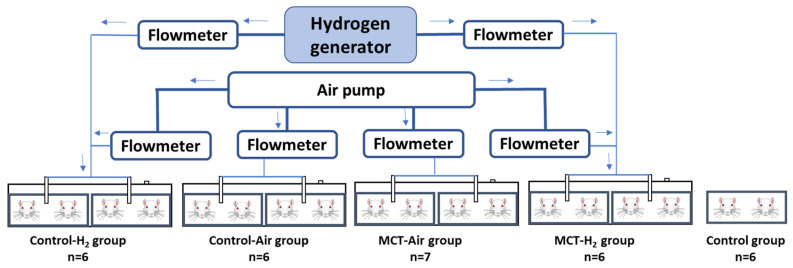
Schematic diagram of the experimental setup. *Hydrogen generator*—hydrogen generator producing hydrogen in such quantities that the final concentration in the air mixture is 4%. *Air pump*—pump to supply air to the chambers containing the animals. *Flowmeter*—flowmeter to control the amount of hydrogen and air entering the animal chambers.

After receiving an injection of either MCT or its solvent, the animals were assigned to five experimental groups. Two groups inhaled only atmospheric air (MCT-Air, *n* = 7; Control-Air, *n* = 6), while two other groups inhaled atmospheric air supplemented with 4% H_2_ (MCT-H_2_, *n* = 6; Control-H_2_, *n* = 6) (Figure 1). Hydrogen or air inhalation was performed twice daily for 2 h each, with a 2 h interval between sessions, over a period of at least three weeks following MCT or solvent administration. Jin et al. in similar designed experiments in rats have shown that two 90 min inhalations of 5% hydrogen a day ameliorated oxidative stress and glucose metabolism disorder in the brain [18].

An additional intact control group (Control, *n* = 6) was maintained under standard vivarium conditions throughout the experiment. This group was included to exclude chamber effects on baseline physiology. The MCT-Air, MCT-H_2_, Control-Air, and Control-H_2_ groups were housed in specialized ventilated containers with a controlled supply of either atmospheric air or air enriched with 4% hydrogen.

#### 2.1.4. Experimental Design

Animals were kept in plastic 130 L containers (SAMLA 203.764.41, Inter IKEA Systems, Delft, The Netherlands). Two T2 animal cages were placed in each container, with 4 rats in each cage. An air compressor (Hiblow XP 40, Techno Takatsuki Co., Osaka, Japan) was used to ventilate the containers. Hydrogen was supplied from a H_2_ generator (Pioner, Vodorodpomogaet, Moscow, Russia). The supply of hydrogen and atmospheric air was controlled by rotameters (LZB-3, LZM-4T, Hefei, China). It was 0.15 L/min for hydrogen and 4 L/min for air. The hydrogen content in this case was 3.5–4.0% in the air breathed by the rats (Figure 1). The duration of the experiment was 21 days. The experimental setup and design were developed by us and were validated in our previous study [7].

#### 2.1.5. Hemodynamic and Baroreflex Parameters Measurement in Awake Animals

**Figure 2:**
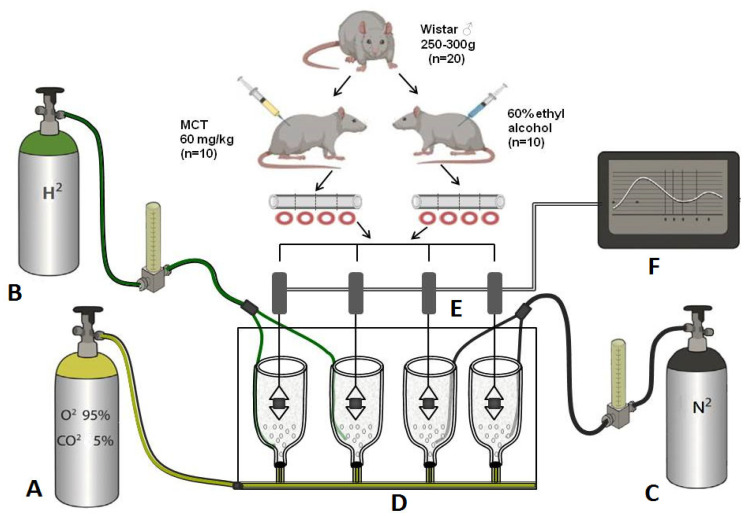
Study design and the scheme of the experimental setup. (**A**)–Gas mixture of oxygen (95%)/carbon dioxide (5%), (**B**)–Hydrogen 50 ppm, (**C**)–Nitrogen 100%, (**D**)–Four-chamber organ bath with Krebs solution and rat aortic rings, (**E**)—Force displacement transducer, (**F**)–Data acquisition system (PowerLab) and computer.

Before the start of the experiment, systolic blood pressure (SBP) was repeatedly measured in all animals, both experimental and control groups, using the plethysmographic method with the LGraph program (LGraph E14-140, Moscow, Russia), and the rats were stratified to ensure comparable baseline SBP between the groups (Figure 2). In addition, SBP was measured weekly throughout the experiment in all groups. On the 20th day of the experiment, plastic catheters were implanted into the femoral vein and artery under zoletil-xylazine anesthesia (xylazine hydrochloride 6 mg/kg (Xyla, JSC “Interchemi Werk”, Püünsi, Estonia), tiletamine 17.5 mg/kg, zolazepam 17.5 mg/kg (Zoletil^®^ 100, Valdepharm, Incarville, France)) in rats with MCT-PH or control groups. After surgery, rats were housed in individual cages in the same inhalation boxes. Hydrogen inhalation was performed in the same mode as during the whole experiment (2 h inhalation, 2 h rest, 2 h repeated inhalation daily). One day after catheter implantation, i.e., on day 22 after injection of MCT or its solvent, hemodynamic parameters were measured in awake animals by the direct method using a Statham sensor (Statham Instrument Inc., Oxnard, CA, USA). Mean blood pressure (MBP), systolic blood pressure (SBP), diastolic blood pressure (DBP) and heart rate (HR) were measured. We also assessed the BP response to the administration of the hypotensive NO donor sodium nitroprusside (NP, 4.5 μg/kg in 30 μL 0.9% NaCl (DiaM, Moscow, Russia) and the hypertensive agent α_1_1-adrenomimetic phenylephrine hydrochloride (PE, 2 μg/kg in 30 μL 0.9% NaCl, (Sigma-Aldrich, Schnelldorf, Germany)). Both drugs, PE and NP, were injected intravenously, and the duration of the BP and HR responses were within 15–20 s, as was shown in the classical paper by Coleman et al. [19]. To calculate the baroreflex coefficient (BRC), we calculated the deviation of mean BP from baseline and the change in HR as BRC = ΔHR/ΔMBP.

#### 2.1.6. Hemodynamics in Anesthetized Animals

Immediately after registration of hemodynamic parameters in awake animals, rats were anesthetized with urethane (MP Biomedicals, Irvine, CA, USA,1.2 g/kg, intraperitoneally) and again MBP, SBP, DBP, and HR, as well as right ventricular systolic pressure (RVSP), were measured by direct method, which was necessary to confirm MCT-PH development.

To measure RVSP, a polyethylene catheter (CJSC “Medsil”, Mytishchi, Russia) was inserted through the jugular vein into the right ventricle and left in place for 10–15 min. The previously implanted femoral arterial catheter was used to measure systemic hemodynamic parameters.

At the end of the experiment, the animal was euthanized by decapitation under deep anesthesia and the heart removed for morphometry. The right ventricular (RV) index of the heart was calculated as the RV weight divided by the sum of the left ventricular (LV) and septal weights, in percent. Left lung tissue was removed and placed in 10% formalin solution (pH 7.4) for 2–3 days for further morphological studies.

#### 2.1.7. Morphological Analysis

Following standard sample preparation procedures [20] serial 5-μm thick sections were cut from the lung paraffin blocks for staining with haematoxylin and eosin and Giemsa solution for mast cells (MCs) identification and 2-μm thick sections for immunohistochemistry. MCs’ tryptase secretion was determined immunohistochemically using mouse monoclonal antibodies against tryptase (Abcam #ab2378, dilution 1:2000). Bound mouse primary antibodies were detected with AmpliStain™ Horseradish Peroxidase (HRP) conjugates (SDT GmbH, Baesweiler, Germany) according to manufacturers’ instructions. The HRP label was visualized using the DAB substrate kit (Vector Laboratories, Burlingame, CA, USA). The sections were counterstained with hematoxylin. Immunopositive cells were quantitatively analyzed in 30 fields of view at ×20 magnification. All histological sections were analyzed in a blinded fashion using a Zeiss Imager.A2 microscope (Carl Zeiss Microscopy GmbH, Oberkochen, Germany), QuPath v0.5.0 software (University of Edinburgh, Edinburgh, UK) [21].

### 2.2. Isolated Aorta Studies

#### 2.2.1. Design of the Experiment

This experiment involved two groups of animals—rats with MCT-PH and a control group. On day 21 after administration of MCT or its solvent the rats were euthanized (600 mg/kg chloralhydrate (ChemMed, Moscow, Russia)), the thoracic aorta was isolated and the vascular responses to vasoconstrictors and vasodilators were tested. Experiments were performed on four aorta rings in parallel using a 4-chamber organ bath. Two rings were assessed in the presence of hydrogen, and the other two served as nitrogen controls (control for the H_2_ effect). Nitrogen, as an inert gas control, was included to account for potential non-specific effects associated with bubbling a gas through the physiological solution and was administered for this purpose in a volume equivalent to that of hydrogen. This protocol also served to maintain consistent dissolved oxygen levels [22]. The oxygen concentration in the solution, monitored with an oximeter (Expert-001, Econix-Expert, Moscow, Russia), was maintained at 12–13 ppm by adjusting the flow of a 95% O_2_/5% CO_2_ gas mixture. Nitrogen was used as an inert gas, in the same volume as hydrogen, in order to balance the oxygen level, since there is evidence that when the hydrogen level in the solution increases, the oxygen level may decrease [22]. Hydrogen concentration was verified with a methylene blue-based assay system (H_2_Blue, MiZ Company, Kanagawa, Japan) and maintained at 0.2–0.3 ppm. Study design and the scheme of the experimental setup are shown in Figure 2.

Thus, 4 experimental groups of vessels were obtained: MCT, MCT-H_2_, Control, Control-H_2_. The testing protocols were the same in all experimental groups.

#### 2.2.2. Aortic Ring Preparation

The rats were anesthetized with intraperitoneal injection of chloralhydrate overdose (600 mg/kg) and euthanized by decapitation. The chest was rapidly opened, and the descending thoracic aorta was dissected and immersed in ice-cold Krebs bicarbonate buffer solution (118.2 mM NaCl, 4.6 mM KCl, 2.5 mM CaCl_2_, 1.2 mM KH_2_PO_4_, 1.2 mM MgSO_4_, 24.8 mM NaHCO_3_, and 10.0 mM glucose). The preparations were cleaned of excessive connective tissue, cut into 4 ring segments 3 mm in length and placed into 10 mL chambers (four-chamber organ bath, Hugo Sachs Elektronik, March-Hugstetten, Germany) containing Krebs solution, which was maintained at 37 °C ± 0.5 °C and aerated continuously with a gas mixture of 5% CO_2_ and 95% O_2_. The pH of the solution was kept at 7.40 ± 0.05. Each aortic ring was mounted between two stainless steel hooks. The upper hook was connected to the force displacement transducer (K30; Hugo Sachs Elektronik, March-Hugstetten, Germany) and changes in isometric force were recorded using PowerLab 8/35 data acquisition system (AD Instruments, Bella Vista, Australia) and software LabChart 7 Pro (AD Instruments, Bella Vista, Australia) (Figure 2) [23,24].

#### 2.2.3. Experimental Protocol

The aortic rings were initially equilibrated for 60 min with a resting tension of 2.0 g. At the start of each experiment, the rings were contracted with 30 mM KCl to validate the contractile function and were then washed 3 times with fresh bathing media every 15 min. The presence of intact endothelium in vascular preparations was confirmed by relaxation responses to 3 µM acetylcholine in phenylephrine-pre-contracted (0.3 µM) rings. After complete washout of acetylcholine and phenylephrine from the organ bath, when the tone returned to its initial level, the responsiveness of the aortic rings to different substances (phenylephrine, acetylcholine and sodium nitroprusside) in the presence and absence of hydrogen was assessed. All substances were cumulatively added in increasing concentrations. Each concentration of substances was added only when the response to the previous dose was stable. The substances were administered sequentially after complete washing of the vessels from the previous substance. Bubbling of the chambers with hydrogen and nitrogen commenced 20 min before testing the responses of the rings to each substance and turned off before washing [24].

To study the changes in sensitivity to α_1_-adrenergic receptor agonists, the aortic rings were exposed to cumulative concentrations of phenylephrine (0.001–30 µM). To study the effect of hydrogen on endothelium-dependent and endothelium-independent vascular relaxation, phenylephrine-pre-contracted (submaximal concentration of phenylephrine (0.3 µM) was used) aortic rings were treated with cumulative concentrations of acetylcholine (0.003–30 µM) and sodium nitroprusside (0.001–3 µM), respectively.

#### 2.2.4. Drugs

The following drugs were used: monocrotaline (Sigma Aldrich, Darmstadt, Germany), acetylcholine chloride (ACROS Organics, Fair Lawn, NJ, USA), L-phenylephrine chloride and sodium nitroprusside (Sigma Chemical Co., St. Louis, MO, USA). Stock solutions were made by dissolving the compounds in distilled water. All solutions were freshly prepared before use and protected from light.

#### 2.2.5. Bioethical Approval

The research protocol was approved by the Bioethics Committee of Moscow State University No. 11.5-sod, dated 18 January 2024.

#### 2.2.6. Data Analysis

Statistical analysis was performed using GraphPad Prism 10.0 (GraphPad Software Inc., San Diego, CA, USA). Data from isolated aortic ring experiments were analyzed as contractile responses of the aortic rings to increasing concentrations of phenylephrine and were presented as percentages of the maximal response induced by 30 mM KCl. Maximum contractile responses to KCl and phenylephrine were expressed as developed tension (g). The −lgEC_50_ (negative logarithm of effective concentration which produced 50% of maximum response to KCl) and maximum response were calculated. Relaxation responses to acetylcholine and sodium nitroprusside were expressed as the percentages relative to the pre-contraction induced by phenylephrine (0.3 µM). In both cases, the −lgEC_50_ (the negative logarithm of the concentration at which the ring relaxed to 50% of its initial contraction) and the maximum relaxation were calculated. Emax = 100% indicates complete reversal of phenylephrine pre-contraction.

Quantitative data are presented as mean ± standard deviation (M ± SD), mean ± SEM (standard error of the mean) of n experiments. Normality of distribution was tested using the Shapiro–Wilk test.

To investigate differences between two independent samples, Student’s *t*-test was used for normal distribution, Mann–Whitney test for non-normal distribution, and paired comparisons were performed using paired *t*-test and Wilcoxon W-test. For comparison of multiple samples, one-way ANOVA was used for normal distribution and Kruskal–Wallis test for non-normal distribution.

Two-way analysis of variance (two-way ANOVA) was used to analyze the influence of two factors, taking into account their interaction under conditions of normality of distribution. Where data were unsuitable for analysis in their original form, log transformed values were used, followed by a repeated normality check and analysis of variance on the transformed data. When normality could not be achieved through transformation, the Aligned Rank Transform ANOVA (ART-ANOVA) was used.

Statistical outliers were excluded using the ROUT criterion with Q not > 1%. Differences were considered statistically significant at *p* < 0.05.

## 3. Results

### 3.1. Study of the Dynamics of Systemic Blood Pressure During 21 Days

**Figure 3:**
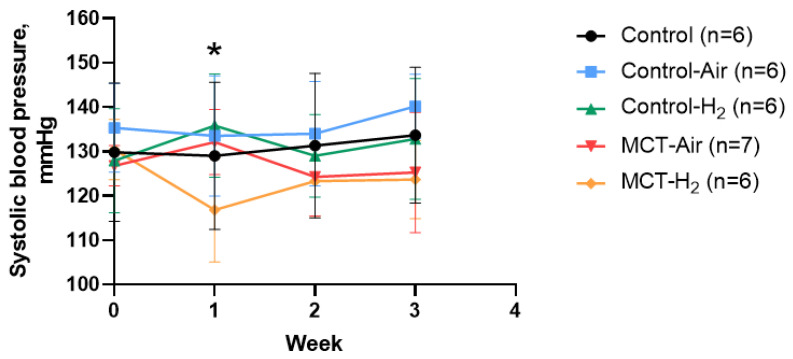
Change in systolic blood pressure in awake animals of all experimental groups during 21 days. * 1 week, MCT-Air vs. MCT-H_2_, *p* < 0.05, two-way ANOVA.

Measurement of systemic BP one week after the start of the experiment showed a mean decrease in systemic BP in the MCT-H_2_ group of 13.6 ± 9.6 mmHg (130.4 ± 6.8 mmHg before the experiment and 116.8 ± 11.7 mmHg at the end of the first week, *p* = 0.07), which was not observed in the other experimental groups, and these differences persisted, although to a lesser extent, until the end of the experiment. This decrease was not statistically significant. However, compared with the systemic BP value in the MCT-Air group, the difference was 15.3 mmHg and reached statistical significance (Figure 3, *p* < 0.05).

### 3.2. Awake Animal Study

**Figure 4:**
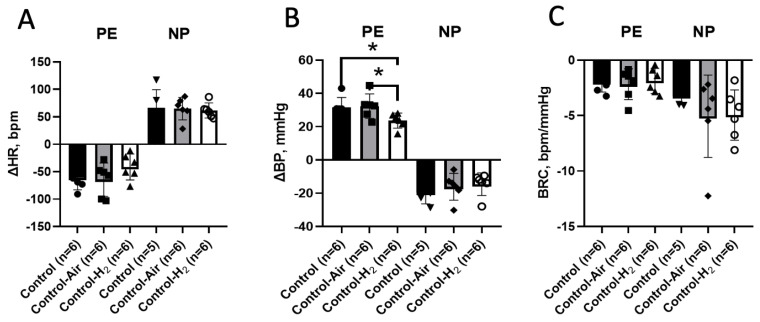
Changes in mean blood pressure, heart rate and baroreflex ratio, in response to administration of PE and NP to awake control group rats. (**A**) HR response; (**B**) MBP response; (**C**) BRC. * Control-H2 vs. Control, Control-Air, *p* < 0.05, one-way ANOVA.

On day 22, the hemodynamic parameters were measured in awake animals of all experimental groups. Bolus administration of PE during baroreflex testing in the control groups caused an increase in mean AP (MBP) of 32.3 ± 7.4 and 23.7 ± 4.5 mm Hg in the Control-Air and Control-H_2_ groups, respectively (Figure 4B). This resulted in a reflex decrease in HR of 65 ± 30 beats/min in the Control-Air group and 42 ± 24 beats/min in the Control-H_2_ group (Figure 4A). In the Control-H_2_ group, there was a less pronounced increase in MBP in response to PE administration, with an average of 8.6 mmHg (*p* < 0.05). The reciprocal decrease in HR was less evident, but not significant, and there was no significant difference in calculated BRC (−2.2 ± 1.4 and −1.9 ± 1.1 between the values of the Control-Air and Control-H_2_ groups, respectively, Figure 4C) (*p* < 0.05).

**Figure 5:**
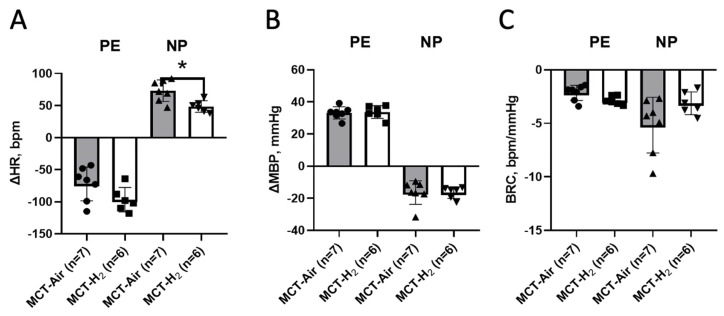
Changes in mean blood pressure, HR and BRC in response to PE and NP administration to awake rats of MCT-Air and MCT-H_2_ groups. (**A**) HR response; (**B**) MBP response; (**C**) BRC. * MCT-Control vs. MCT-H_2_, *p* < 0.05, Mann–Whitney test.

Intermittent inhalation with the 4% hydrogen during PE administration resulted in a similar increase in BP in both MCT-PH groups, which was 33.2 ± 3.8 mmHg in the MCT-Air group and 33.7 ± 4.0 mmHg in the MCT-H_2_ group (Figure 5B). At the same time, HR decreased in the MCT-H_2_ group to an average of 97 ± 19 beats/min, while a decrease of 72 ± 26 beats/min was observed in the MCT-Air group breathing only atmospheric air (*p* > 0.05, Figure 5A).

With a similar BP reduction response to NP administration (16.4 ± 7.4 mmHg in the MCT-Air group and 16.5 ± 3.7 mmHg in the MCT-H_2_ group), we observed a statistically significant reduction in HR response in the MCT-H_2_ group (Figure 5A, 49 ± 9 beats/min vs. 73 ± 17 in the MCT-Air group, *p* < 0.05). This resulted in a decrease in BRC NP (Figure 5C, −3.1 ± 1.1 in the MCT-H_2_ group vs. −5.2 ± 2.6 in the MCT-Air group, *p* < 0.05).

According to the data obtained, MBP (Figure 5B) was not statistically different in the Control-Air and Control-H_2_ groups and was 117 ± 9 mmHg in two groups. No statistically significant differences were found between the groups for SBP, MBP and HR (Table 1).

Thus, the mode of intermittent H_2_ inhalation used does not cause statistically significant differences in key hemodynamic parameters in awake animals.

### 3.3. Study of Hemodynamic Parameters in Anesthetized Animals

**Figure 6:**
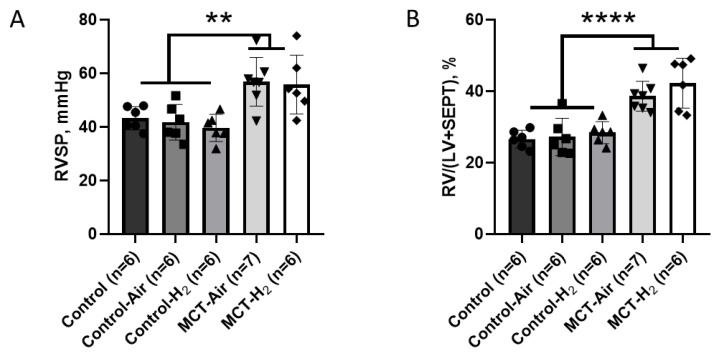
Indices of pulmonary hypertension in rats. (**A**) Right ventricular systolic pressure (RVSP); (**B**) index of RV hypertrophy. Control groups vs. MCT groups ** *p* < 0.01, **** *p* < 0.0001, one-way ANOVA.

Both groups administered monocrotaline developed pulmonary hypertension three weeks after its administration, as evidenced by an increase in right ventricular systolic pressure (RVSP) and right ventricle (RV) hypertrophy (Figure 6). Hydrogen inhalation had no effect on RVSP (Figure 6A) and RV hypertrophy (Figure 6B). The RVSP and RV hypertrophy index were 56.4 ± 9.6 mmHg and 40.3 ± 5.7% in the groups with MCT, and 41.5 ± 5.3 mmHg and 27.4 ± 3.6% in the control groups, with *p* < 0.01 for RV pressure and *p* < 0.0001 for RV hypertrophy index.

Examination of the values of MBP, systolic BP (SBP) and diastolic (DBP) in the anesthetized animals of all experimental groups revealed no significant differences in these parameters between them (Table 1). However, there was a significant decrease in HR in the MCT-H_2_ group compared to the MCT-Air group (419 ± 45 beats/min and 466 ± 34 beats/min, respectively). No differences in HR were found in the control groups (Table 1).

Thus, 3 weeks after, the administration of monocrotaline at a dose of 60 mg/kg after pulmonary hypertension was developed in rats. Intermittent inhalation with atmospheric air with of 4% hydrogen in the mode “2 h inhalation–2 h pause–2 h inhalation”, as well as chronic action of H_2_, does not affect such parameters of PH as RVSP and the index of RV hypertrophy. At the same time, in anesthetized animals with MCT-H_2_ in comparison with the MCT-Air group, there is a significant decrease in HR in contrast to awake rats.

### 3.4. Morphological Analysis

**Figure 7:**
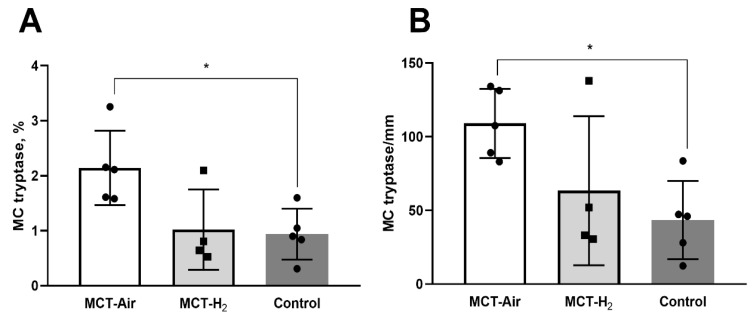
The amount of MCs tryptase (**A**) in % and (**B**) per mm of histological section of lung tissue, MCT-Air vs. Control * *p* < 0.05 one-way ANOVA.

According to the results of tryptase-containing MCs detection, the number of MCs/1 mm^2^ area and MCs % were significantly lower in the healthy control group than in the MCT group: 36.7 ± 16 MCs/mm^2^ and 1.0 ± 0.5% versus 109.0 ± 23.4 MCs/mm^2^ and 2.4 ± 0.7% (Figure 7). Despite the apparent decrease, the MCT-H_2_ group was not statistically different from the other two groups.

**Figure 8:**
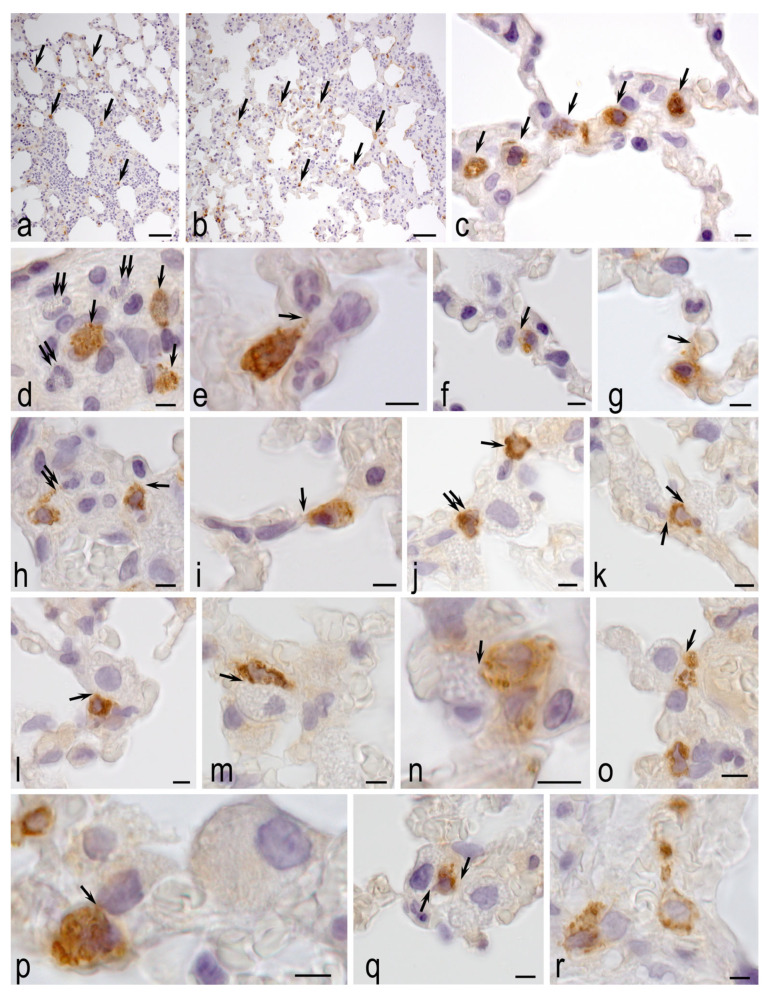
Immunohistological detection of MC tryptase in the lungs of MCT-Air group animals on day 22. (**a**,**b**) Signs of acute pneumonia with areas of total infiltration of alveoli and interalveolar septa with segmented neutrophilic leukocytes, microatelectasis and dystrophic changes in alveolocytes. High levels of MCs (arrow). (**c**) Large number of mucosal MCs in the interalveolar septum (arrow). (**d**) Paracrine co-localization of MCs (arrow) and neutrophilic granulocytes in the stroma of the lung airways. (**e**) Involvement of MC in transendothelial migration of neutrophils from the microcirculatory channel into the stroma of the interalveolar wall, interaction (arrow). (**f**,**g**) Effects of MC tryptase (indicated by arrow) on neutrophil granulocytes. (**h**) Secretion of tryptase into the capillary endothelium of the respiratory alveolar wall (indicated by arrow) and neutrophil (double arrow). (**i**) MCs secrete tryptase to type I alveolocytes (arrow). (**j**) Tryptase-mediated intercellular signaling to the basal membrane of capillary endothelium (arrow, top) and granular leukocyte (arrow, bottom) and to two type II alveolocytes (double arrow). (**k**–**q**) Morphological equivalents of active MC tryptase exposure to type II alveolocytes (indicated by arrow). (**r**) Evidence of active tryptase secretion into the microcirculatory bed of the respiratory lung section. Scale bar: 5 µm.

The revealed changes in the lung tissue of animals in the MCT-Air group (Figure 8), such as infiltration with neutrophils and dystrophic changes in alveolocytes, confirm the active participation of MCs, including tryptase secretion, in the development and intensification of the severity of inflammatory processes. Intensive migration and local accumulation of mucosal MCs in interalveolar septa and their interaction with neutrophils indicate one of the key roles of these cells in the intensification of inflammation during the development of MCT-induced pulmonary hypertension (Figure 8).

**Figure 9:**
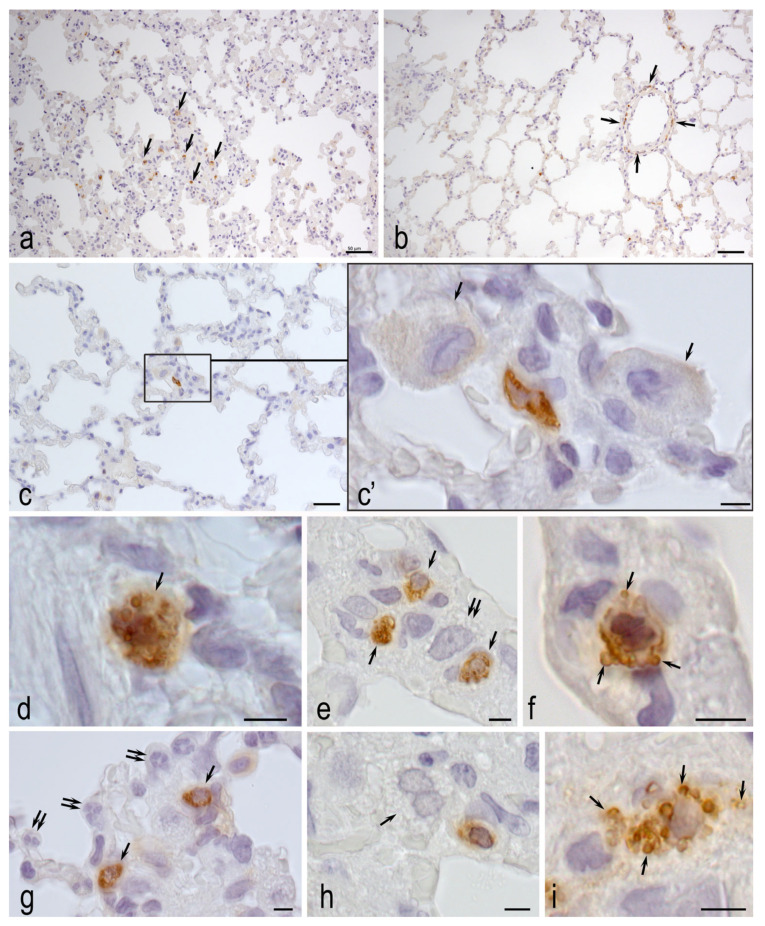
Immunohistological detection of MC tryptase from lung MCs of animals in the MCT-H_2_ group on day 22. (**a**) Preservation of MC-rich loci (indicated by arrow). (**b**) Preferential location of MCs around the vein (indicated by arrow). (**c**) Individual MC in the respiratory part of the lung (**c’**)—magnified fragment. (**c**) MC located between two type II alveolocytes (indicated by arrow). (**d**) MC in the adventitia of a large bronchus (indicated by arrow). (**e**) MC in the wall of structural components of the respiratory tract (indicated by arrow), one of which is adjacent to a type II alveolocyte (indicated by double arrow). (**f**) Active secretion of tryptase as part of granules to targets in the extracellular matrix of the wall of the structural components of the acinus of the respiratory tract (indicated by arrow). (**g**) Migration of MCs (indicated by arrow) and neutrophils (indicated by double arrow) in the stroma of the respiratory lung. (**h**) Paracrine interaction between MCs and type II bi-nuclear alveolocytes. (**i**) Release of tryptase-positive secretory granules into the extracellular matrix. Scale bar: a–c 50 μm, others 5 μm.

In the MCT-H_2_ group (Figure 9), MCs remained present in certain lung areas, indicating that H_2_ inhalation did not fully suppress monocrotaline-induced inflammation. However, H_2_ significantly reduced the intra-organ MCs population, particularly mucosal-type cells near respiratory acinus structures, suggesting an anti-inflammatory effect that counteracts monocrotaline’s pro-inflammatory action. MCs were predominantly located around veins, implying a role in microcirculation and vascular responses, and were also found in the adventitia of large bronchi and near type I/II alveolocytes, highlighting their involvement in the air–blood barrier. Their secretion of proteases like tryptase influences tissue remodeling and inflammation, and their close interaction with type II alveolocytes points to key paracrine regulatory mechanisms in the lung’s local microenvironment.

**Figure 10:**
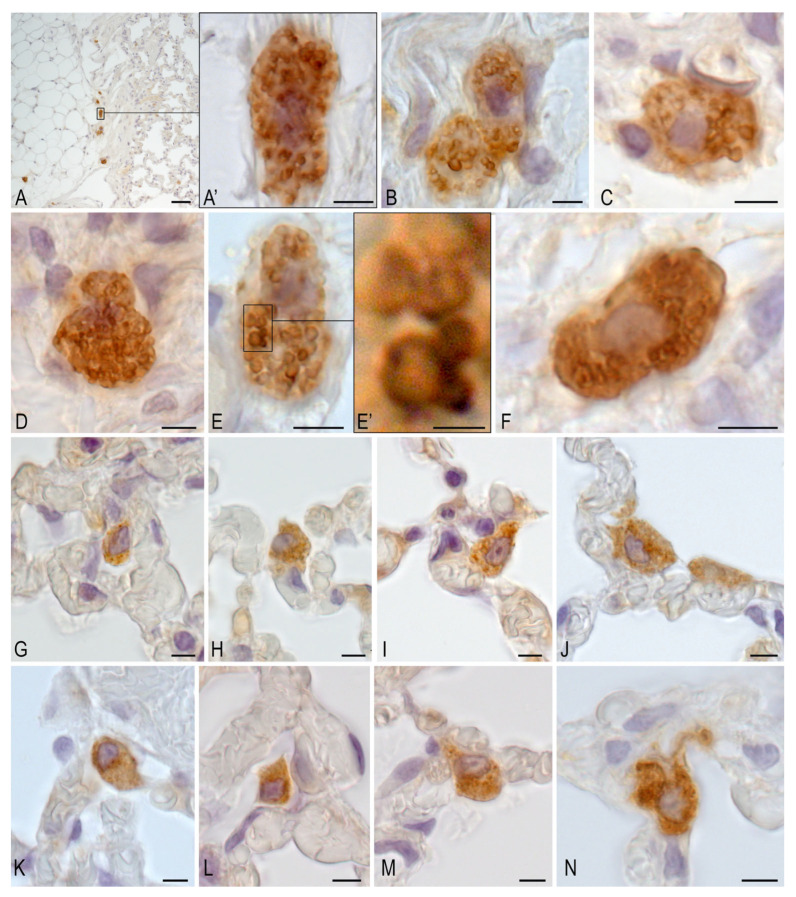
Immunohistological tryptase detection of pulmonary MCs from control group animals on day 22. (**A**–**F**) MCs of connective tissue type accompanying large intra-organ vessels and airways (bronchi). (**A**) Predominant localization of large MC in the bronchial adventitia. (**A’**) Enlarged fragment. (**A**) Large secretory granules are located in the cytoplasm of MC. (**B**) Two touching MCs, unevenly filled with large secretory granules. (**C**) MCs with a well-defined nucleus and secretion of a whole granule into the extracellular matrix. (**D**) MCs completely filled with secretory granules. (**E**) MC with secretory granules of different sizes. (**E’**) Magnified fragment (**E**). A large mature granule in a state of exchange of secretome components with smaller immature granules. Tryptase is located at the periphery of the granules. (**F**) Granule-filled MC with no evidence of whole granule secretion. (**G**–**N**) Subpopulation of mucosal MCs accompanying structural components of the respiratory lung. Varying arrangements of small MCs in the stroma of the walls of respiratory bronchioles and alveoli, almost universally in contact with capillaries. Some MCs have elongated sections of cytoplasm oriented towards targets of the tissue microenvironment (**M**,**N**). Scale bars: A—50 µm, E’—1 µm, the rest—5 µm.

In healthy control rats, tryptase-positive MCs were present in normal numbers and divided into two subtypes: connective tissue MCs (around large bronchi and vessels) and mucosal MCs (in bronchioles and alveoli, often near capillaries). Their morphology and positioning suggest active roles in tissue maintenance and intercellular communication under physiological conditions (Figure 10).

Thus, in the results of the analysis of the tissue microenvironments of the lungs of animals, in the group of MCT-H_2_, the signs of decrease in the intra-organ populations of tryptase-positive MCs in the structural components of bronchial tree and respiratory section of lungs against the background of decreases in changes in the pathological character were noted, which indicates a protective role of hydrogen in acute inflammation. The shown effect suggests the novel mechanisms of formation of the positive therapeutic effects of hydrogen inhalation.

### 3.5. Heart Hypertrophy in Rats with MCT-PH

In the group of rats injected with MCT, it significantly increased the relative RV mass (*p* < 0.05) compared to the control groups, which confirms the development of PH (Table 2).

### 3.6. Isometric Tension Experiments on Isolated Aorta Rings

**Figure 11:**
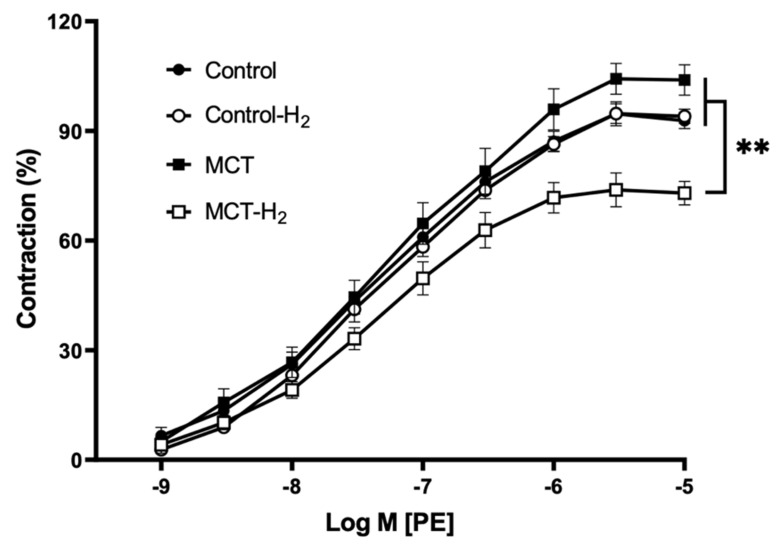
Concentration–response curves for phenylephrine in thoracic aorta rings isolated from the control and MCT-PH rats. Each point represents the mean value from 10 rats ± SEM. Contractions induced by 30 mM KC1 were taken as 100%; ** MCT-H_2_ vs. Control, Control-H_2_, MCT, *p* < 0.01, two-way ANOVA test.

**Figure 12:**
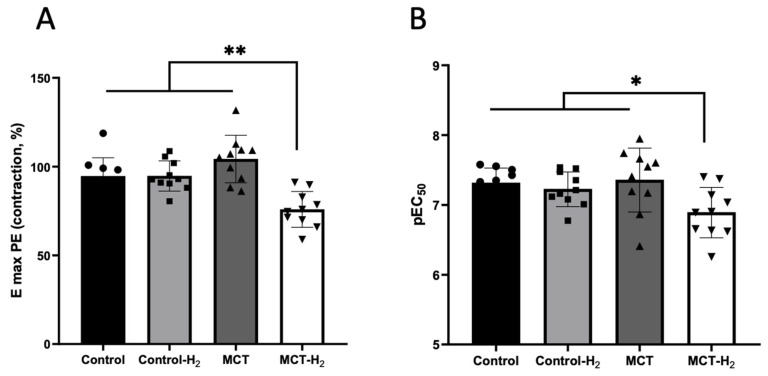
(**A**) The −lgEC_50_ values and (**B**) the maximum contraction (Emax) induced by phenylephrine (as a percentage of the maximum effect of 30 mM KCl administration). MCT-H_2_ vs. other groups, * *p* < 0.05, ** *p* < 0.01, one-way ANOVA.

Phenylephrine elicited a concentration-dependent contraction of rat aortic rings in all tested groups. The maximum contractions were obtained at a concentration of 3 µM phenylephrine. The concentration–response curves of phenylephrine-induced contractions are shown in Figure 11. Vessels of rats with monocrotaline pulmonary hypertension preincubated with hydrogen (MCT-H_2_) were less sensitive to the contractile effect of phenylephrine compared to all other groups (Figure 12A). The −lgEC_50_ value of phenylephrine and the maximal contractile response in group MCT-H_2_ were significantly lower than in the other studied groups (Figure 12B).

There were no significant differences in phenylephrine-induced maximal tension between rats with monocrotaline-induced pulmonary hypertension (MCT) and rats of the control groups (Control, Control-H_2_) (Figure 11, Table 2).

**Figure 13:**
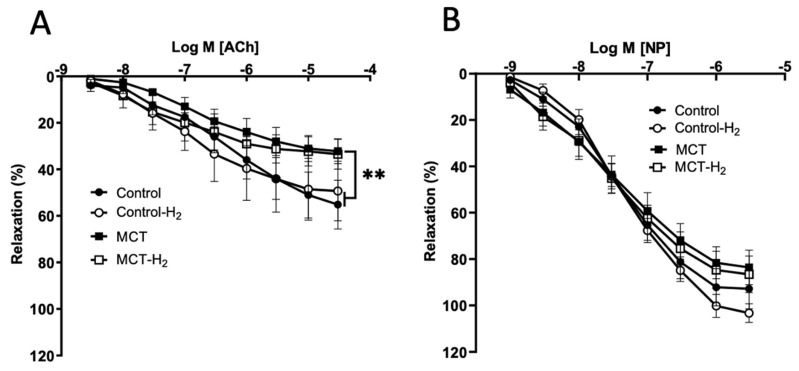
Relaxation responses of isolated aortic rings of control and MCT-PH rats. Vasorelaxant effect of (**A**) acetylcholine and (**B**) sodium nitroprusside on phenylephrine-induced contraction. ** MCT vs. Control, Control-H_2_, *p* < 0.01, two-way ANOVA test.

Acetylcholine (0.003–30 µM) and sodium nitroprusside (0.001–3 µM) caused dose-dependent relaxation in all groups’ PE pre-contracted (0.3 µM) aortic rings (Figure 13A,B).

Acetylcholine-induced endothelium-dependent relaxation of aortic rings was significantly reduced in the MCT group compared to control vessels. Meanwhile, the acetylcholine dose–response curve for the MCT-H_2_ group showed no statistically significant difference when compared to either the control groups or the MCT group (Figure 13A).

Endothelium-dependent relaxation of aortic rings induced by acetylcholine did not differ between the MCT-H_2_ and control groups, whereas the response to acetylcholine was significantly reduced in the MCT group compared to control vessels (Figure 13A). There were no significant differences in the vasodilating effect of sodium nitroprusside on the vascular segments of the studied experimental groups (Figure 13B).

Preincubation with H_2_ (MCT-H_2_ group) reduced the sensitivity of the aorta of rats with pulmonary hypertension to the contractile action of PE, without affecting endothelium-independent relaxation mediated by NP. Although a non-significant trend toward modulating endothelium-dependent relaxation was observed in the MCT-H_2_ group, this interpretation is limited by the lower baseline of PE-induced precontraction in this group, a factor known to influence the amplitude of the relaxant response.

## 4. Discussion

In our study, we used an intermittent H_2_ inhalation protocol similar to that used in human clinical trials (two sessions of 2 h with a 2 h interval) for 3 weeks [6]. However, this exposure did not result in a statistically significant reduction in systemic BP compared with baseline BP values (Figure 3). It is noteworthy that at the end of the first week of the experiment there was a significant reduction in SBP in the MCT-H_2_ group compared to the MCT-Air group, but this effect was not maintained in the following weeks of the experiment.

In contrast to studies demonstrating a sustained hypotensive effect with chronic use of H_2_ [7], our results can be explained by an insufficient duration of intermittent inhalation. This assumption is supported by data from a large retrospective clinical study (1182 patients in hydrogen inhalation group, 1182 patients in control group) in which a significant reduction in blood pressure was observed in patients with hypertension only after 24 weeks of continuous therapy [25]. The authors emphasize that long-term hydrogen therapy can be considered as a promising antihypertensive approach in clinical practice, demonstrating a statistically significant improvement in BP parameters compared to the control group.

MCT-PH is a well-established experimental model of pulmonary hypertension associated with interstitial lung injury characterized by the development of inflammatory and fibrotic changes [26]. In our previous studies, we demonstrated that chronic exposure to 4% H_2_ in animals with MCT-induced hypertension resulted in a significant 30% reduction in the number of tryptase-positive MCs compared to the MCT control group, indicating a pronounced anti-inflammatory effect of H_2_. This property of H_2_ is well documented in the literature for various models of ischemia–reperfusion injury in the heart, brain and kidney [25]. However, in the context of pulmonary hypertension, the anti-inflammatory effect of hydrogen against MCs was first described in our work [7].

One of the possible pathways for the implementation of the biological effects of molecular hydrogen may be associated with the regulation of MC activity [27]. In the present study, we continued the investigation of the tryptase-containing MCs population in the lung tissue of rats with MCT-PH. The data obtained showed that MCT administration caused a significant increase in the number of MCs in the MCT-Air group to 109.0 ± 23.4 MCs/mm^2^ compared to control values (36.7 ± 16 MCs/mm^2^; *p* < 0.05). Although the total number of MCs in the MCT-H_2_ group did not differ statistically from the MCT-Air group, no differences from the healthy animal group were observed either. The application of H_2_ against the background of monocrotaline action resulted in a tendency to decrease the number of MCs, and their level did not differ from the indicators of healthy animals. As shown by Russell et al., H_2_ selectively suppresses the formation of hydroxyl radicals and peroxynitrite, which may explain the observed decrease in MCs degranulation and its damaging effect on alveolocytes in the H_2_ inhalation group. Reduced MC degranulation activity attenuates the inflammatory response by decreasing the release of histamine into the tissue microenvironment. This correlates with the reduced secretion of exosomes that modulate the immune process [28]. In addition, the ability of H_2_ to inhibit NF-κB-mediated (nuclear factor kappa B) immune cell activation identified by the authors is consistent with the reduction in tryptase-dependent inflammation in our study [23]. These results confirm that even intermittent inhalation of H_2_ has a pronounced anti-inflammatory effect comparable to that of chronic hydrogen application. The data obtained allow us to consider the dynamics of the MCs population as a promising marker of the efficacy of hydrogen therapy in various cardiovascular pathologies.

In addition to studying the main parameters of the cardiovascular system (BP and HR) in experiments to investigate the effect of intermittent inhalation of H_2_ in rats with MCT-PH, we studied the BP and HR responses of these animals to the administration of hypertensive and hypotensive substances and the BRC. The arterial baroreflex is one of the key mechanisms of nervous regulation of hemodynamics. The influence of the baroreflex in the regulation of heart rate is manifested in a decrease in heart rate in response to an increase in BP and, conversely, in an increase in heart rate in response to a decrease in BP, which ensures the maintenance of cardiovascular homeostasis. Baroreflex regulation of BP is achieved by balancing sympathetic and parasympathetic influences on HR and vascular resistance. The analysis of the role of sympathetic and parasympathetic influences on the changes in HR was not the focus of our study. Two series of experiments were carried out to investigate the possible effect of H_2_ on the mechanisms of blood pressure regulation: on control animals and on rats with MCT-PH. In both cases, groups of animals were used that breathed only atmospheric air or with 4% hydrogen supplementation, in addition to a vivarium control. When the α_11_-agonist phenylephrine was administered, there was a significant reduction in the hypertensive response in the Control-H_2_ group, which was accompanied by a reduction in the HR response and did not affect the BRC (Figure 5). These data suggest that H_2_ may reduce the sensitivity of peripheral adrenoreceptors to sympathetic influences. No differences in blood pressure responses to either phenylephrine or sodium nitroprusside were found between the monocrotaline groups. However, the change in HR in response to the administration of the hypotensive agent sodium nitroprusside was significantly smaller in the hydrogen-breathing MCT-H_2_ group than in the MCT-Air group. The magnitude of the baroreflex decreased from 5.2 ± 2.0 to 3.1 ± 1.0. A similar effect was observed in experiments on SHR rats, where the impact of hydrogen-rich water on blood pressure and baroreflex function was investigated. In the absence of an effect on the BP value, the authors found a normalization of the baroreflex value [29]. The attenuated HR response to NP-induced hypotension in the MCT-H_2_ group may underlie the reduction in blood pressure observed after the first week of hydrogen inhalation. A similar mechanism likely contributes to the sustained hypotensive effect seen in this group during chronic exposure to 4% H_2_ in air, as previously reported by our team [7].

In our previous paper with intravenous administration of the α_11_-agonist phenylephrine, we suggested that H_2_ might reduce the sensitivity of peripheral adrenergic receptors to sympathetic influences [30]. To test this hypothesis, an additional series of experiments was performed on an isolated rat aortic preparation with and without MCT-PH. This method allowed the influence of neurohumoral mechanisms of vascular tone regulation to be excluded. It also eliminates factors influencing hydrogen bioavailability in vivo, allowing controlled concentrations to be produced in the solution wash vessels and the direct effect of H_2_ on vascular reactivity to be assessed. H_2_ was added to the perfusion medium at a concentration of 0.2 to 0.3 ppm (in preliminary studies this concentration was found to maintain adequate H_2_ levels and not lead to hypoxia). A dose–response study of phenylephrine administration showed a significant reduction in constriction in aortic preparations from rats in the MCT group incubated with H_2_, confirming the ability of H_2_ to reduce the sensitivity of α_1_-adrenergic receptors to adrenergic agonists. The observed modest improvement in endothelium-dependent relaxation in the MCT-H_2_ group is difficult to interpret unambiguously, as the modulating influence of H_2_ resulted in a lower baseline level of PE-induced preconstriction in this group. It is important to emphasize that since we could not have anticipated the modulating effect of hydrogen on phenylephrine sensitivity a priori, and it was not evident during the experiments, the protocol employed standard phenylephrine doses to induce preconstriction prior to vasodilator testing. Therefore, this effect warrants further investigation using a modified experimental approach.

Intermittent inhalation of H_2_ following a clinically relevant protocol did not produce a sustained hypotensive effect on systemic blood pressure. Baroreflex testing revealed an attenuated heart rate response to sodium nitroprusside-induced hypotension in the MCT-H_2_ group, while responses to phenylephrine remained unchanged. In isolated aortic ring experiments, H_2_ reduced vascular sensitivity to the α_11_-adrenoceptor agonist phenylephrine, as evidenced by the decreased maximal contractile response. Endothelium-dependent relaxation responses to acetylcholine in vessels from MCT-H_2_ rats did not reach statistical significance compared to either the control groups or the MCT group. Although these results point toward potential autonomic effects of hydrogen, the study did not include direct measurements of sympathetic or parasympathetic activity; therefore, addressing this question through targeted autonomic recordings will be an important objective for future research.

The absence of a systemic hypotensive effect with intermittent inhalation can be explained by insufficient exposure to reach therapeutic concentrations in the heart and vascular centers of the brain. The data obtained by Yamamoto et al. [31] on the uneven distribution of hydrogen in the organs (maximum concentration in the liver, minimum concentration in the kidneys, delayed saturation of the muscles) are particularly important for the interpretation of our results. Thus, the effect on pulmonary MCs that we observed indicates a high sensitivity of the lung tissue to H_2_. This supports the hypothesis of a predominantly local effect of inhaled hydrogen in MCT-PH, which requires further investigation of the mechanisms of its accumulation in lung tissue [31].

We speculate that the inhibition of the HR response by hydrogen to the decrease in BP by NP could facilitate the effects of hypotensive drugs in arterial hypertension that require experimental validation.

This manuscript represents a final revision without additional experiments or reanalysis. The findings should be considered hypothesis-generating and require confirmation through direct measurements of autonomic activity and additional mechanistic studies. The study has certain limitations and was conducted on a limited number of animals, and in vitro experiments utilized a minimal set of vascular preparations, which constrains the generalizability of the conclusions. In addition, the applied methods characterize integrated physiological responses rather than the underlying molecular or autonomic mechanisms. Nevertheless, the overall pattern of the data indicates biologically meaningful trends that warrant further investigation using larger cohorts and more mechanistically targeted approaches.

## 5. Conclusions

The use of intermittent H_2_ inhalation in our work imitating the protocol of human studies revealed their moderate effect on the state of the cardiovascular system and lung tissue under MCT-PH. However, when studying the baroreflex regulation of BP, we assume that a decrease in the HR response to the decrease in BP is in favor of a possible effect of H_2_ on the central structures of baroreflex regulation. In addition, it has been shown that H_2_ can reduce the sensitivity of vascular adrenoreceptors to adrenergic agonists. Morphological analysis showed that hydrogen inhalation did not change the total number of MCs, but modified their distribution and activity. There was a decrease in cell infiltration in the respiratory compartments and a decrease in tryptase secretion, indicating a partial anti-inflammatory effect of H_2_.

## Figures and Tables

**Table 1:** Hemodynamic parameters of awake and anesthetized rats.

	Control	Control-Air	Control-H_2_	MCT-Air	МCТ-Н_2_
	*n* = 6	*n* = 6	*n* = 6	*n* = 7	*n* = 6
Awake animals					
HR, beats/min	369 ± 36	379 ± 37	395 ± 35	431 ± 34	423 ± 32
MBP, mmHg	112.6 ± 13.6	116.3 ± 12.2	117.0 ± 6.0	108.5 ± 10.9	110.5 ± 4.6
SBP, mmHg	137.4 ± 15.7	135.2 ± 17.4	144.6 ± 7.8	131.1 ± 11.8	127.0 ± 4.6
DBP, mmHg	92.5 ± 12.7	99.1 ± 11.0	94.4 ± 6.3	89.0 ± 10.4	95.3 ± 7.9
Anesthetized animals					
HR, beats/min	429 ± 32	429 ± 26	430 ± 33	466 ± 34	419 ± 45 *
MBP, mmHg	104.1 ± 11.4	79.0 ± 17.2	84.3 ± 20.1	98.0 ± 12.6	103.7 ± 6.2
SBP, mmHg	126.8 ± 12.5	96.9 ± 18.7	110.7 ± 24.6	118.0 ± 14.1	122.4 ± 9.9
DBP, mmHg	89.9 ± 11.9	66.8 ± 16.7	69.8 ± 19.8	84.0 ± 12.2	87.7 ± 4.9

* MCT-H_2_ vs. MCT-Air, *p* < 0.05, one-way ANOVA.

**Table 2:** Mass and indicators of right ventricular hypertrophy in rats on day 21 of the experiment.

Group	*n*	RV Mass, g	RV/Heart	RV/LV + S	(RV/Body Mass) × 1000
Control	10	0.14 ± 0.03	0.18 ± 0.02	0.26 ± 0.03	0.49 ± 0.06
MCT	10	0.21 ± 0.04 *	0.52 ± 0.04 *	0.40 ± 0.08 *	0.77 ± 0.15 *

* Control vs. MCT control vs. MCT, *p* < 0.05, one-way ANOVA.

## Data Availability

Data are available on request from the authors.
